# COVID19 epidemic outbreak: operating rooms scheduling, specialty teams timetabling and emergency patients' assignment using the robust optimization approach

**DOI:** 10.1007/s10479-022-04667-7

**Published:** 2022-05-10

**Authors:** Mojtaba Arab Momeni, Amirhossein Mostofi, Vipul Jain, Gunjan Soni

**Affiliations:** 1 Jam Faculty of Engineering, Persian Gulf University, Bushehr, Iran; 2 Wellington School of Business and Government, Victoria University of Wellington, Wellington, New Zealand; 3 Malaviya National Institute of Technology Jaipur, Jaipur, India

**Keywords:** Operating rooms scheduling and assignment, Timetabling of specialty teams, Coronavirus pandemics, Stochastic-base robust optimization approach

## Abstract

The health care system is characterized by limited resources, including the physical facilities as well as skilled human resources. Due to the extensive fixed cost of medical facilities and the high specialization required by the medical staff, the problem of resource scarcity in a health care supply chain is much more acute than in other industries. In the pandemic of the Coronavirus, where medical services are the most important services in communities, and protective and preventive guidelines impose new restrictions on the system, the issue of resource allocation will be more complicated and significantly affect the efficiency of health care systems. In this paper, the problem of activating the operating rooms in hospitals, assigning active operating rooms to the COVID-19 and non-COVID-19 patients, assigning specialty teams to the operating rooms and assigning the elective and emergency patients to the specialty teams, and scheduling their operations is studied by considering the new constraints of protective and preventive guidelines of the Coronavirus. To address these issues, a mixed-integer mathematical programming model is proposed. Moreover, to consider the uncertainty in the surgery duration of elective and emergency patients, the stochastic robust optimization approach is utilized. The proposed model is applied for the planning of operating rooms in the cardiovascular department of a hospital in Iran, and the results highlight the role of proper management in supplying sufficient medical resources effectively to respond to patients and scheduled surgical team to overcome the pressure on hospital resources and medical staff results from pandemic conditions.

## Introduction

World health organization has defined the health system as a system containing all activities whose main purposes are to maintain, promote, and restore health (World Health Organization, 2009). Besides health promotion, the availability of health services and the lack of differences between individuals in providing services or justice in services are new paradigms of the health system.

Hospitals are scarce resources of health care systems that play an essential role in improving patients' health. Due to the lack of hospital resources in terms of equipment and specialized teams, proper planning is essential for the effective use of hospital resources. Also, planning models of hospitals should be able to provide appropriate solutions to deal with uncertain events such that the solutions not only meet the essential needs but also are effective in terms of cost and resource utilization. In this paper, a mixed-integer model is proposed for addressing some of the most challenging issues in the hospitals, including activation of hospitals' operating rooms, the assignment of specialty teams to working days of the planning horizon (Time-table of specialty teams), the assignment of active hospitals to specialty teams, the assignment of emergency patients to specialty teams and the scheduling of patients, both elective and emergency ones. The model is further extended to a robust scenario-based model to take into account the uncertainty in the number and treatment time of emergency patients.

In a timetable of staff, it should be specified in what time blocks a coordinated team of a particular specialty should be presented at the hospital. An incorrect timetable can lead to overtime costs and inefficiency of hospital services. Also, staff may request repeated changes to the proposed schedule if their interests are not included in the program (Sadler, 2015). Hence, an appropriate timetable can both increase the operational efficiency of hospitals and lead to greater employee satisfaction. The design of a suitable timetable that meets the hospital's commitments and requirements is one of the aims of the paper, while this was regarded as a predetermined issue in many of the previous papers.

The scheduling problem of patients' surgery in the operating rooms determines the assignment of patients to the operating rooms in the working days and the sequence of their surgeries. This is a complex problem that involves the organization of various stakeholders, resources, and services. Specialist physicians, nurses, anesthesia teams, medical equipment, and resuscitation beds should be taken into account in an integrated manner. Also, Lee et al. (2017) acknowledged that a well-designed arrangement of the problem should consider the well-being of all staff and also prioritize the allocation of available resources efficiently and effectively.

Due to the high cost of activating the operating rooms, it is cost-effective for hospitals to activate operating rooms as few as possible. However, reducing the number of operating rooms increases patients' waiting times, which can have serious health consequences. Hence, there is always a trade-off between increasing the service level of hospital care and reducing costs in determining the number of active operating rooms. Such trade-off can be established by considering tardiness for patients' waiting time or binding constraints in the mathematical representation of the problem. For example, in Chile, when the waiting time of patients exceeds a predetermined maximum waiting time, a voucher should be paid to them to cover their additional expenses for referring to private hospitals (Barrera et al., 2020). On the other hand, when there is uncertainty in the parameters, a scenario-based robust model can not only establish such a trade-off by considering penalties for violating the constraints in different scenarios but also could control the risk-taking level of decision-makers in terms of model feasibility. In this regard, the robust optimization approach in the present study, which is described in detail below, booster uncertain models for planning the operating rooms.

Clearly, without knowing the referring patients or patients who are scheduled to go to the hospital, it is not possible to optimally determine what number of operating rooms should be open on working days or what specialty teams are required to be present each day. In other words, the mentioned issue should be considered in an integrated manner to provide an optimal and coordinated solutions. Although, there are papers, which deals with one or some of these issues, however, not all of them are considered in an integrated model. Therefore, the integrated model proposed in this paper addresses all mentioned issues, and offers a more efficient solution based on which, different parts of the hospital interact with others more efficiently and effectively.

**Fig. 1:**
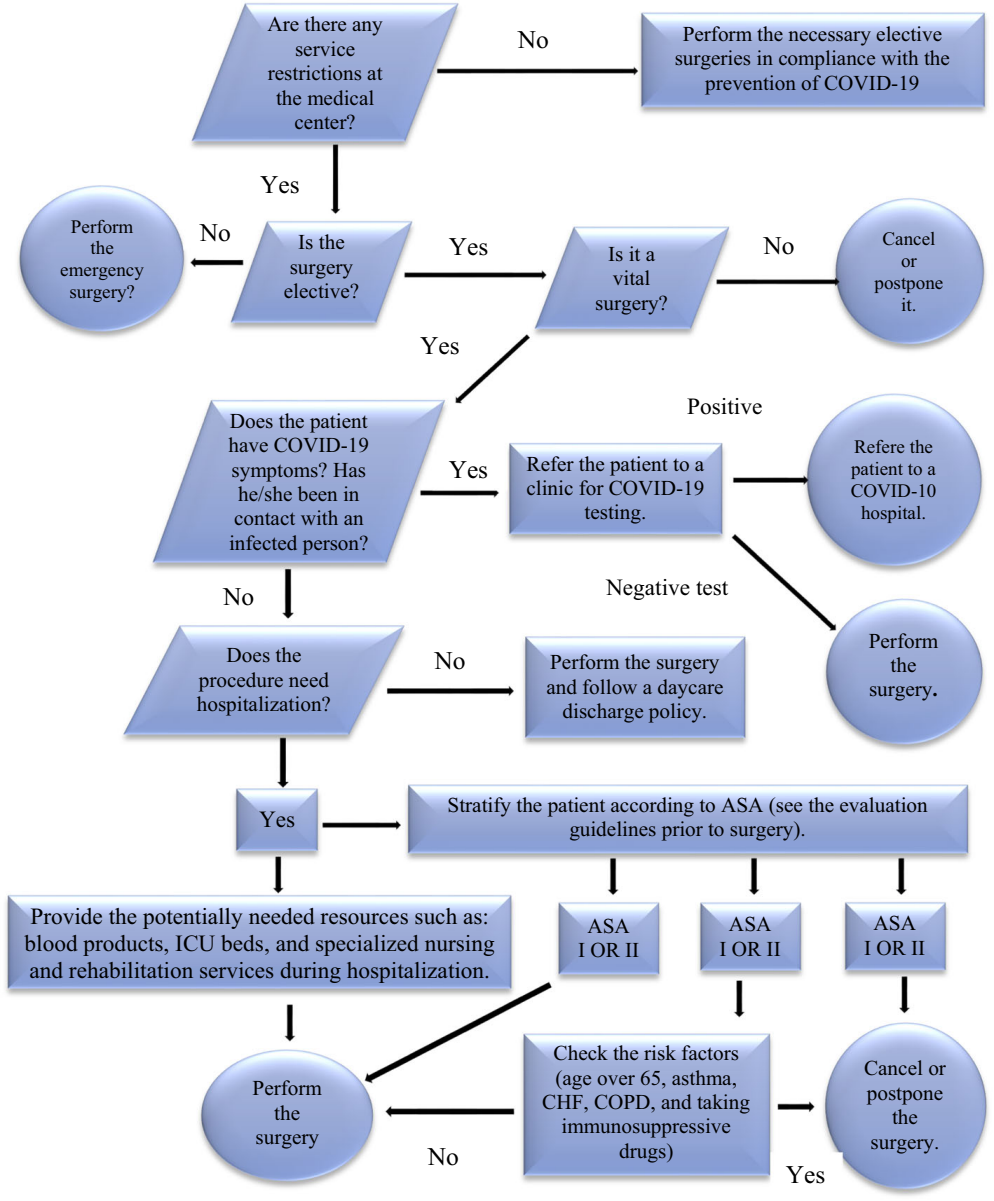
Risk Stratification algorithm of performing elective surgeries during the COVID-19 pandemic

Another point is about the additional restrictions imposed on hospitals during epidemics and pandemics such as Coronavirus (COVID-19), which alters the usual planning and scheduling of operating rooms. For example, the Ministry of Health and Medical Education (MOHME) of Iran has notified all affiliated hospitals (public and private) to separate the wards of COVID-19 patients from the non-COVID-19 ones. Moreover, the surgeries of elective patents have been categorized as "Essential Elective Surgeries," "Semi-Essential Elective Surgeries," and "Not-Essential Elective Surgeries." While hospitals are allowed to operate on emergency patients, the surgery of elective patients is only allowed for the category of "Essential Elective Surgeries." The flowchart of this instruction has been depicted in Fig. 1 Besides these instructions, there are some suggestions by MOHME that could be taken into account in setting the timetable of treatment staff. For example, the sequential assignment of a specialty team to working days and accommodation of the team at the predetermined location of the hospital during the nights are advised by hospitals. In this case, COVID-19 screening tests of staff are not necessary every day, and staffs have enough time to rest in an environment far enough away from the hospital that is more contaminated than other environments due to patient visits. Taking into account the above considerations, the proposed model presented is different from previous models and presents the concerns raised during the pandemics in the resulting solutions. The proposed model is in line with the need for an urgent call of operation research (OR) and other operations-related fields of academic studies that cover the COVID-19 issues and challenges (Choi, 2021; Queiroz & Fosso Wamba, 2021).

## Literature review

The operating rooms are scarce resources of hospitals. The greater demand for operating rooms than their supply leads to negative consequences such as long delays, stresses on the patients and their families and reduced hospital visits by the patient, and as a result, loss of income (Stepaniak & Pouwels, 2017). These are management challenges that entail the optimization of operating room functions regarding a low waiting time for patients. The assignment should balance between low use of operating rooms or their low efficiency and high use and excessive erosion of them (Strum et al., 1998). Indeed, the highest costs in surgical care are operating room costs and the wages of surgical teams (Macario et al., 1995), and most of these costs are operating room labor costs (Dexter et al., 1999). Therefore, a robust and efficient system that ensures the productivity of human resources and reduces the cost of operating rooms is essential.

There are three approaches for the scheduling of operation rooms, including open scheduling, blocked scheduling, and semi-blocked scheduling. In the first approach, the patients are operated based on the arrival order. This entails high experts of operating room managers in predicting the request for surgery and does not take into account the cost consideration of the rooms. The second approach assigns an operating room to a specialty team in each working day such that no other specialty could use the room even if the room is idle (Patterson, 1996). To increase the efficiency of this approach, a proper estimation of the arrival rate of emergency patients as well as a suitable program for calling the elective patients of surgical teams are essential (Miller et al., 2014). The third approach both assigns a number of rooms to some specialties and considers the rest for unforeseen patients to undergo surgery in the order of their arrival. Another modification of this approach is the use of an assigned operation room by other surgical teams if the intended team is idle (Patterson, 1996). Given the circumstances in which limited surgery is allowed in pandemic COVID-19 conditions, and most surgeries are predetermined, the second approach is used in the present study.

Furthermore, as mentioned in Abedini et al. (2017), there are three levels for scheduling the operating rooms. The first level is the strategic level in which the type of surgeries and the medical staffs employed in the hospital for a long-term time horizon are determined. For example, the allocation of operating rooms to surgical teams in a one-year planning horizon is studied at this level. Given the low accuracy of estimates over long periods, some researchers have questioned the appropriateness of such models' results (Dexter et al., 1999; Guerriero & Guido, 2011; Masursky et al., 2008). In the second level, namely the tactical level, the Master Surgical Schedule (MSS) is presented. The number and type of operating rooms, the working hours of them as well as the assignment of surgery teams to the rooms in a planning horizon of 1 week to 3 months are recognized in MSS. For reasons such as equipment failure, unavailability of medical staff due to recess, and official holidays, tactical-level decisions may be subject to change and require corrections (Guerriero & Guido, 2011). Hence, operational-level decisions, which are daily or weekly, determine the executive instructions in detail and modify strategic and tactical decisions as appropriate. Some researchers, such as Jebali et al. (2006) and Testi et al. (2007), have proposed models that involve more than one level of decision making. The proposed model of the paper is a tactical-level decision support tool in the scenes that determines the timetable of surgical teams. On the other hand, the model addresses many operational decisions, such as the time and sequence of surgeries done by the teams. In this regard, the proposed model is more similar to the tactical model of M'Hallah and Visintin (2019), which had a 2-week planning horizon and addressed the decisions related to the Master Surgical Schedule.

Many academic studies of planning the operation rooms and scheduling have provided deterministic mathematical models. These models are valuable in terms of introducing objective functions and functional constraints of hospitals and operating rooms. Denton et al. (2006) presented a model to minimize the waiting times of surgeries to determine the sequence of the start time of surgeries. The problem of scheduling and allocation of operating rooms was investigated by Cardoen et al. (2010) regarding deterministic surgery times. The proposed model was presented as an expert computer system, and the results of its implementation showed that it performed better than the conventional manual system of the studied hospital. Aringhieri et al. (2015) presented an integrated two-level model for the planning and scheduling of operating rooms. The objective function of their model was the minimization of the waiting cost of patients and the operational costs of hospitals. In doing so, both the utility of patients as well as the efficiency of hospitals are taken into account in the optimal solutions. Also, proving the complexity of the problem, a two-level meta-heuristic algorithm was proposed to extract the solutions. Durán et al. (2017) examined the performance of two optimization models and two algorithms for scheduling patients in operating rooms by taking into account their relative priorities. They showed that their proposed methods improve operating room utilization rates from 10 to 15% in comparison to the current manual techniques. Hooshmand et al. (2018) proposed a three-level model for evaluating the problem of scheduling and rescheduling of operating rooms. They introduced the rescheduling time as a variable that should be decided endogenously as one of their contributions. A dynamic operating room scheduling model was offered by Zhu et al. (2020). In that model, the assignment of operating rooms to specialties, the assignment of operating rooms to surgeons, and the identification of Surgical sequence were explored. To solve the model, a hybrid meta-heuristic of Grey Wolf Optimizer (GWO) with Variable Neighborhood Search (VNS) was proposed. The presented model of the paper, in line with the mentioned model, minimizes costs, including the cost of waiting for patients, the cost of overtime for surgical teams, as well as the costs associated with activating operating rooms as a function of the target.

Recent studies have focused on the uncertain models to better reflect real-world conditions. Choi and Wilhelm (2014) investigated the duration of surgical assistants in hospitals and the sequence of their use in operating rooms. To solve the problem, the modified Newsvendor model by the objective function of minimizing the earliness and tardiness times are utilized. Wang et al. (2014) studied the problem of scheduling and assignments of the operating rooms by considering uncertainty in the operating times and the emergency demands and stipulating the possibility of canceling surgeries. The same research was conducted by van den Berg et al. (2014) by additionally assuming that the arrival time of patients is stochastic as well. Some researchers, such as Lee and Yih (2014) and Addis et al. (2015), used the robust and fuzzy approach to solve the uncertain problem of scheduling and assignment of operating rooms. Saadouli et al. (2015) scrutinized the assignment of surgeries to operating rooms considering uncertain operating times and patient resuscitation time. Razmi et al. (2015) analyzed the planning of both emergency and elective patients, presuming that there is uncertainty in the need for unique and rare equipment. The assignment of patients to hospitals, the activations of operating rooms in the selected hospitals, and the assignment of patients to the working days of hospitals were explored in Roshanaei et al. (2017). Rachuba and Werners (2017) proposed a fuzzy multi-criteria model for providing a robust operating room schedule assuming stochastic surgery times. They investigated the assignment of both emergency and elective patients to days and rooms. They also used the benders decomposition approach to solve the problem. Najjarbashi and Lim (2019) explored the uncertainty in the surgery durations by proposing a stochastic mixed-integer linear programming (SMILP) and utilizing the concept of the Conditional Value-at-Risk (CVaR) to minimize the CVaR of overtime and idle time costs. They showed that their approach outperforms the widely used expected value (EV) approach. Kamran et al. (2018) considered the uncertainty in the surgery duration and the arrival time of patients using the two-stage stochastic and two-stage chance-constrained stochastic models. Also, they used the benders decomposition approach the solve the models. To make the proposed model of the paper more in line with the operational conditions of hospitals, the uncertainty in the duration of surgeries is taken into account. Because the probabilistic distribution of durations could be estimated by historical data, the proposed model is a two-stage stochastic model regarding the decisions about operating room activation and the design of timetables as the first stage variables. Also, a robust optimization approach is proposed, which makes a trade-off between the solution feasibility, the solution optimality, and the existence of small and applicable changes in the variable part of the solution for scenarios. In fact, the incorporation of the stochastic and robust approaches, which was less considered in previous research, enables hospital managers to choose a robust solution according to their utility for the mentioned features.

It should be noted that in the hospital processes, usually resources from different departments are involved. Therefore, if the relationship between the sources of different departments is ignored, the solution intended for one department may be suboptimal and not in coordination with other departments. Hence, some operating room scheduling studies have linked the surgical process to other departments, such as Intensive Care Units (ICU). The scheduling problem of operating rooms by considering the limitation of post-anesthesia beds was also researched by Latorre-Núñez et al. (2016). They also incorporated the possibility of the arrival of emergency patients and considered a maximum duration for handling these patients. Jebali and Diabat (2017) not only considered the capacity of the operating rooms in determining the assignment of emergency and elective patients to working days and the sequence of their surgery but also the capacity of the Intensive Care Unit (ICU) as a post-treatment stage of patients after the surgery. They used a two-stage chance-constrained stochastic programming model to consider the uncertainty in the surgery time and patient Length of Stay (LOS) as patients' post-operative in the ICU. Belkhamsa et al. (2018) provided a model for the operating room scheduling problem aimed at minimizing the finish time of the last stage of a surgery process. The stages included pre-operative, intraoperative, and post-operative stages, each of which was subject to capacity constraints. A multi-period and multi-resource scheduling problem of operating rooms were investigated by Vali-Siar et al. (2018). They embodied various resources in their mixed-integer linear programming model, including human resources, equipment, as well as beds in the pre-operative holding unit, recovery unit, ward, and intensive care unit. Also, to take into account the uncertain durations of surgeries and recovery operations, they extended the model to an uncertain model using a robust optimization approach. M'Hallah and Visintin (2019) addressed the Master Surgical Scheduling problem (MSS) in planning the operation rooms at the tactical level. They considered a two-week planning horizon in which the number and type of surgeries were determined. They used Master Surgical Schedule as a reference to assign specialties to the operating rooms. They took into consideration the intensive care unit beds and post-surgery beds beside the operating rooms to provide an integrated model considering the interaction between hospital resources. They aimed to maximize the expected operating theatre's throughput in their stochastic model. Atighehchian et al. (2020) introduced a two-step stochastic approach for scheduling operating rooms to minimize the total idle and overtimes of operating rooms. They also considered the multi-resource requirements of surgeries and the dependency between the resources. Coban (2020) raised another constraint about the resources of operating rooms that was the waiting time for the sterilization of reusable medical devices (RMDs) is other sterilized batches of RMDs are not available. For this purpose, he proposed a mixed-integer linear programming model to minimize the total costs of sterilization, postponement, and makespan. In the present paper, considering that patients, especially COVID-19 patients, need intensive care after surgery, and during the corona pandemic, the capacity of the intensive care unit is very limited and sensitive, scheduling operating rooms is explored in connection with the subsequent resuscitation processes.

The robust optimization approaches are widely used to tackle problems with uncertain parameters. This approach can not only provide robust and feasible solutions with high probability regarding the realization of uncertain parameters but can also take into account the degree of risk-taking of decision-makers to balance between the optimality and feasibility of solutions. There are several categories for robust optimization models. However, a highly relevant category to justify the proposed model of the paper is based on the level of available information and is "deterministic," "probabilistic," and "possibilistic" (Mostofi et al., 2020). When only the interval of uncertain parameters, without any more information such as the probabilistic distribution, can be estimated, the deterministic type is applicable. For this type, the proposed approaches of Bertsimas et al. (2004) and Mostofi et al. (2020) could be referred to. If the probabilistic distribution of uncertain parameters is available or could be estimated using historical data, the probabilistic approach based on the probabilistic theory is raised. Methods in this context are the extension of well-known stochastic models such as moment information (Kang, 2008), two-stage optimization approach (Assavapokee et al., 2008), multi-stage optimization approach (Bertsimas et al., 2008), risk theory (Bertsimas & Takeda, 2015) to the robust optimization concept, and maxmin approach (Bai et al., 2022). The possibilistic type approach has been developed to deal with uncertainty in the form of fuzzy numbers or Linguistic variables (Alberto Campos et al., 2006). The proposed model of the present paper is related to the post-pandemic period when daily statistics are collected and trends can be extracted. Hence, a probabilistic type robust optimization approach is used to take into account the uncertainty in the surgery duration of elective and emergency patients as well as the resuscitation time of them in the hospital. This robust approach is well-suited for providing quick and effective responses in critical situations such as pandemics (Yang et al., 2021). Moreover, as explained later, the model is capable of considering the penalty of exceeding the regular operating time and proceeding with the remaining planned surgeries in the overwork times. Finally, the model can suggest solutions that in different scenarios, there is little difference in the hospital operating plan.

Based on the review of literature, some research gaps which justify the need of the present research are as follows. There is a need to model the emerging dynamic COVID-19 situations to capture the intricacies and efficacies of the operating table scheduling and assignments. Further, there is a strong urge to develop a robust optimization model, which penalizes any deviation from hospital resource capacities and provide valuable insights to dynamics in specialty team timetabling and emergency patients assignments, which is very useful in managing hospital operations under COVID-19 pandemic. Further, in line with recent studies in the context of hospital scheduling methods, the proposed model takes into account the dependency between different resources. Although this dependency, as well as partitioning the hospital ward to the COVID-19 and COVID-19 wards, intensifies the capacity constraints and activates more hospital facilities such as operating rooms in the planning horizon, it makes the solution more responsive to the incoming critical conditions of the pandemic. Finally, given the large number of possible scenarios, which might exist in reality, the proposed robust optimization model manages the uncertainties associated with possible scenarios in an efficient cost-effective way.

## Model

In this section, the deterministic model of the paper, considering the expected assumptions in the pandemic condition, is presented. Next, it is explained how the model could be generalized to a robust stochastic programming model to account for the uncertainty in demand and operating time, and post-operating resource usage of emergency patients.

According to Fig. 1, the new preventive guidelines for surgical procedures in the new conditions categorized patients needing surgery into elective and emergency patients. An elective patient has already met a specialist who prescribes surgery for him or her to go to a hospital on a specific day to undergo surgery according to the schedule set by the hospital managers. Emergency patients, on the other hand, are patients who urgently need surgery, but although their surgery is preferable, the operating room is not pre-planned for them, although it can be anticipated. As shown in Table 1, the surgery of elective patients can be divided into three categories based on the need for surgery: essential, semi-essential, and non-essential.

**Table 1:** The classification of elective patients' surgery

Category	Clinical description	Waiting time for admission	Examples
Essential	Have the potential to get worse as quickly as possible or even become urgent	30 days	Amputation surgery, Heart valve replacement, …
Semi-essential	Causes pain, dysfunction, or disability and is unlikely to get worse quickly	90 days	Colposcopy, …
Not-essential	Can cause pain, dysfunction, or disability, and is unlikely to get worse quickly. They do not have the potential to become urgent	365 days	Cosmetic surgery, …

The elective patients in the "Essential" category refer to laboratories or clinics before going to the hospital to be determined whether they have Coronavirus disease or not. If their tests are positive, they are referred to referral hospitals intended for COVID-19 patients. Otherwise, they get permission to be operated on in the hospital, and the hospital will be informed about their status to decide when they could undergo surgery.

Due to the prevalence of the COVID-19 disease epidemic, some hospitals, which are referred to as referral hospitals, provide services only to COVID-19 patients (emergency or elective with essential surgery) while their staffs have sufficient preventive protections. On the other hand, other hospitals that we refer to them as public hospitals can perform the surgery of elective patients with deemed essential surgery according to a predetermined schedule. Also, these hospitals should admit emergency patients, whether with COVID-19 disease or not, as soon as possible. In the present paper, the scheduling problem for a public hospital in the pandemic of Coronavirus is investigated. For this purpose, some operating rooms in the hospital are considered for the emergency patients who suffered from the Coronavirus disease as well as their disease for which they visit the hospital urgently. Other hospital rooms will serve the elective and emergency patients without Coronavirus disease. The same approach is used to segment the intensive care units (ICU) and post-anesthesia rooms as other stages of the surgery process. Hence, the whole surgical process of patients from their admission to discharge from the hospital is as follows:

1. Upon admission to the hospital, if a patient is in an emergency, first, it is assumed that the patient is corona-positive. Then, in parallel with intensive care, special for COVID-19 patients, diagnostic tests, such as molecular tests, are performed to confirm or reject the assumption about the COVID-19 disease of patients. If the test is positive, the patient is transferred to the operating rooms of COVID-19 patients; otherwise, it is assigned to the operating rooms of non-COVID-19 patients.
2. According to the scheduling model of paper and considering the resource constraints of the hospital; patients are operated on COVID-19 or non-COVID-19 operating rooms according to the schedule set in the operating rooms. Emergency surgical patients with COVID-19 disease must be transferred to ICU units after surgery. Other patients will be moved to ICU units if this is prescribed by the ASA standards, or they will be transferred to post-anesthesia rooms until they will be discharged from the hospital by the diagnosis of a specialist doctor.

Similar to many operating room scheduling problems, it is assumed that there is uncertainty in the surgery duration of emergency patients and the resource usage duration of them, especially for ICU beds. On the other hand, several scenarios are considered for the possible realizations of the uncertain parameters such that each uncertain parameter has a known value in each scenario. To give priority to patients with the worse condition, a health status score is considered for each elective patient. For example, the 1-to-5 scale of Roshanaei et al. (2017), which regards higher values for patients with worse health status conditions, could be used for the objective minimization functions.

Before explaining the operating room scheduling model of the paper, the scenario-based robust optimization approach of the paper which is based on the proposed model of Mulvey et al. (1995) is described. To do so, Model (1), as a standard model, is taken into account.

1 $$\begin{aligned} & {Model \, (1)} \\& {Min\,\,f.y + c.x} \\& {s.t.} \end{aligned}$$

2 $$ A.x = b $$

3 $$ B.y + D.x = 0 $$

4 $$ y \in R^{ + } ,\,\,\,\,x \in R^{ + } $$

Regarding the index $\Omega$ for denoting the stochastic scenarios, Model (1) is rewritten as Model (2) for each scenario $\Omega$.

5 $$ \begin{array}{*{20}l} {Model \, \left( 2 \right)} \hfill \\ {Min\,\,z_{\Omega } = f.y + c_{\Omega } x_{\Omega } } \hfill \\ {s.t.} \hfill \\ \end{array} $$

6 $$ A.y = b $$

7 $$ B_{\Omega } .\,y + D_{\Omega } .\,x_{\Omega } = 0 $$

8 $$ y \in R^{ + } ,\,\,\,\,x_{\Omega } \in R^{ + } $$

In Model (2), *y* is the vector of first-stage variables that should be determined before the realization of scenarios and $x_{\Omega }$ is the vector of the second-stage variables when the scenario $\Omega$ will be realized in the second stage. In the scenario-based robust optimization of the paper, three objectives are followed: (1) the minimization of the expected costs (2) the minimization of constraints violations, and (3) the minimization of the deviation between the solutions of each scenario. To satisfy these objectives, the intended robust optimization model will be as Model (3):

9 $$ \begin{array}{*{20}l} {Model\;\left( 3 \right)} \hfill \\ {Min\,\sum\limits_{\Omega } {p_{\Omega } } .\,z_{\Omega } + \lambda .\,\sum\limits_{\Omega } {p_{\Omega } .|\varepsilon_{\Omega } |} + \delta .\,\left( {\sum\limits_{\Omega } {p_{\Omega } \left| {\left( {z_{\Omega } - \sum\limits_{\Omega ^{\prime}} {p_{{\Omega^{\prime } }} z_{{\Omega^{\prime } }} } } \right)} \right|} } \right)} \hfill \\ {s.t.} \hfill \\ \end{array} $$

10 $$ z_{\Omega } = f.y + c_{\Omega } x_{\Omega } \quad \forall \Omega $$

11 $$ A.\,y = b $$

12 $$ B_{\Omega } .\,y + D_{\Omega } \,.\,x_{\Omega } + \varepsilon_{\Omega } = 0\quad \forall \Omega $$

13 $$ y \in R^{ + } ,\,\,\,\,x_{\Omega } \in R^{ + } ,\,\,\varepsilon_{\Omega } \in R\quad \forall \Omega $$

In Model (3), $\lambda$ and $\delta$ are the weights considered for objectives 2 and 3, respectively. Finally, the nonlinear terms in Model (3) could be linearized, as shown in Model (4).

14 $$ \begin{array}{*{20}l} {Model \, \left( 4 \right)} \hfill \\ {Min\,\,\sum\limits_{\Omega } {p_{\Omega } } .\,z_{\Omega } + \lambda .\,\sum\limits_{\Omega } {p_{\Omega } .\left( {\xi_{\Omega }^{ + } + \xi_{\Omega }^{ - } } \right)} + \delta .\,\sum\limits_{\Omega } {p_{\Omega } .\,\left( {z_{\Omega } - \sum\limits_{\Omega ^{\prime}} {p_{{\Omega^{\prime } }} .\,z_{{\Omega^{\prime } }} } + 2.\,\theta_{\Omega } } \right)} } \hfill \\ \end{array} $$

15 $$ z_{\Omega } = f.y + c_{\Omega } x_{\Omega } \quad \forall \Omega $$

16 $$ A.\,y = b $$

17 $$ B_{\Omega } .\,y + D_{\Omega } \,.\,x_{\Omega } + \varepsilon_{\Omega } = 0\quad \forall \Omega $$

18 $$ \varepsilon_{\Omega } = \xi_{\Omega }^{ + } - \xi_{\Omega }^{ - } \quad \forall \Omega $$

19 $$ z_{\Omega } - \sum\limits_{\Omega ^{\prime}} {p_{\Omega ^{\prime}} z_{\Omega ^{\prime}} } + \theta_{\Omega } \ge 0\quad \forall \Omega $$

20 $$ y \in R^{ + } ,\,\,\,\,x_{\Omega } \in R^{ + } ,\,\,\varepsilon_{\Omega } \in R\quad \forall \Omega $$

## The Sample average approximation (SAA)

Due to a large number of possible scenarios, in reality, the sample average approximation method (SAA) is used to solve the problem for a sample of scenarios. In SAA, the upper and lower bounds of the real objective function are determined through a two-step samples generation procedure (Aydin & Murat, 2013): In the first step, the first-stage variables of each sample are identified, and the lower bound of the objective function is measured as the average objective function of the samples. Next, fixing the first-stage variables of each first-stage sample and generating a large enough size sample in the second stage, the upper bound of the objective function is determined in the second stage. To describe the SAA method, the notations as below are taken into account:

**Table:** 

Notation	Description
M	The number of samples indexed by *m* in the first step of SAA
N	The number of scenarios in each sample of the first step of SAA
*S*_*m*_	The set of scenarios in the sample *m* of the first step ($S_{M} = \{ 1,2,...,M\}$)
N'	The number of scenarios in the second step of SAA

Now, regarding Model (4) and the above notations, the SAA method used in this paper is as the following:

SAA procedure of the paper:

**Initialization:** Generate *N* independent scenarios for each sample *m* ($m = 1,2,...,M$).

1. For each sample *m*, find the optimal solution of the following model and save the optimal objective function and the vector of the first-stage variables as $$v^{m*}$$ and $$y^{m*}$$.
  21 $$ \begin{array}{*{20}l} \\[-32pt]
{v^{m} = Min\,\,\frac{1}{N}.\sum\limits_{{\Omega \in S_{m} }} {z_{\Omega } } + \lambda .\,\frac{1}{N}.\sum\limits_{{\Omega \in S_{m} }} {\left( {\xi_{\Omega }^{ + } + \xi_{\Omega }^{ - } } \right)} + \delta .\,\,\frac{1}{N}.\sum\limits_{{\Omega \in S_{m} }} {\left( {z_{\Omega } - \sum\limits_{{\Omega^{\prime } \in S_{m} }} {p_{{\Omega^{\prime } }} .\,z_{{\Omega^{\prime } }} } + 2.\,\theta_{\Omega } } \right)} } \hfill \\ {s.t.} \hfill \\ \end{array} $$
  22 $$ z_{\Omega } = f.y^{m} + c_{\Omega } x_{\Omega } \quad \forall \Omega \in S_{m} $$
  23 $$ A.\,y^{m} = b $$
  24 $$ B_{\Omega } .\,y^{m} + D_{\Omega } .\,x_{\Omega }^{m} + \varepsilon_{\Omega } = 0\quad \forall \Omega \in S_{m} $$
  25 $$ \varepsilon_{\Omega } = \xi_{\Omega }^{ + } - \xi_{\Omega }^{ - } \quad \forall \Omega \in S_{m} $$
  26 $$ z_{\Omega } - \sum\limits_{\Omega ^{\prime}} {p_{\Omega ^{\prime}} z_{\Omega ^{\prime}} } + \theta_{\Omega } \ge 0\quad \forall \Omega \in S_{m} $$
  27 $$ y^{m} \in R^{ + } ,\,\,\,\,x_{\Omega }^{m} \in R^{ + } ,\,\,\varepsilon_{\Omega } \in R\quad \forall \Omega \in S_{m} $$
2. Calculate the lower bound of the main objective function $$\overline{v}^{M}$$ using Eq. 28.
  28 $$ \overline{v}^{M} = \frac{1}{M}.\,\sum\limits_{m = 1}^{M} {v^{m} } $$
3. Generate a new sample with *N*' scenarios.
4. For each sample *m* of the first step, consider $$y^{m*}$$ as the fixed value of the first stage variables, solve the following model, and denote the optimal objective function of the model by $$\hat{v}^{*m}$$.
  29 $$ \begin{array}{*{20}l} {\hat{v}^{m} = Min\,\,\frac{1}{{N^{\prime } }}.\sum\limits_{\Omega \in S^{\prime}} {z_{\Omega } } + \lambda .\,\frac{1}{N}.\sum\limits_{{\Omega \in S^{\prime } }} {\left( {\xi_{\Omega }^{ + } + \xi_{\Omega }^{ - } } \right)} + \delta .\,\,\frac{1}{N}.\sum\limits_{{\Omega \in S^{\prime } }} {\left( {z_{\Omega } - \sum\limits_{\Omega ^{\prime}} {p_{{\Omega^{\prime } }} .\,z_{{\Omega^{\prime } }} } + 2.\,\theta_{\Omega } } \right)} } \hfill \\ {s.\,t\,.} \hfill \\ \end{array} $$
  30 $$ z_{\Omega } = f.y^{m*} + c_{\Omega } x_{\Omega } \quad \forall \Omega \in S^{\prime } $$
  31 $$ A.\,y^{m*} = b $$
  32 $$ B_{\Omega } .\,y^{m*} + D_{\Omega } .\,x_{\Omega }^{m} + \varepsilon_{\Omega } = 0\quad \forall \Omega \in S^{\prime } $$
  33 $$ \varepsilon_{\Omega } = \xi_{\Omega }^{ + } - \xi_{\Omega }^{ - } \quad \forall \Omega \in S^{\prime } $$
  34 $$ z_{\Omega } - \sum\limits_{\Omega ^{\prime}} {p_{\Omega ^{\prime}} z_{\Omega ^{\prime}} } + \theta_{\Omega } \ge 0\quad \forall \Omega \in S^{\prime } $$
  35 $$ x_{\Omega }^{m} \in R^{ + } ,\,\,\varepsilon_{\Omega } \in R\quad \forall \Omega \in S^{\prime } $$
5. Select the best solution of the main problem $$y^{SAA}$$ by $$y^{SAA} = \mathop {\arg \,\min }\nolimits_{{y^{m*} |\,m = 1,2,...,M}} \,\hat{v}_{m}$$ and determine the upper bound of the main objective function $$v^{SAA}$$ by $$v^{SAA} = \mathop {\min }\nolimits_{{y^{m*} |\,m = 1,2,...,M}} \,\hat{v}_{m}$$. It is worth mentioning that $$y^{SAA}$$ is reported as the solution to the SAA method. Also, the smaller the difference between the upper and lower bounds, the better the performance of the SAA method in extracting the solution.

Furthermore, to simulate the samples of scenarios, the Latin hypercube sampling (*LHS*) method, as a well-known method in this regard, is used. To explain this method, suppose *k* numbers of uncertain variables should be specified in each sample. Moreover, if the number of samples is *n*, the sequence of the uncertain variable in each sample will be as Eq. 36, where *x*_ij_ is the *i*th random sample (regardless of the order of the sample generated from the random variable) of the random variable $\tilde{x}_{j}$.

36 $$ \{ x_{11} ,x_{12} , \, \ldots \, , \, x_{1k} \} ,\{ x_{21} ,x_{22} , \, \ldots \, , \, x_{2k} \} ,...,\{ x_{n1} ,x_{n2} , \, \ldots \, , \, x_{nk} \} $$

Next, the Latin hypercube sampling (*LHS*) method is used to simulate random variables (Olsson et al., 2003). In this method, it is assumed that the probability distribution function of random variables is known or that it could be derived from previous samples of the random variables. In this method, the matrix *P* as an *n* × *k* dimensional matrix is used where each of its columns contains a randomly generated permutation of integers from 1 to *n.* Furthermore, *R* is regarded as a matrix with the same dimension as *P,* whose elements are uniformly generated real numbers in the interval [0, 1]. Using *P* and *R*, the matrix *S* is formed as Eq. 37.

37 $$ S = \frac{1}{N}(P - R) $$

Bearing in mind the matrix *S*, the *i*th sample of *j*th random variable is derived using Eq. 38, where $F_{{x_{j} }}^{ - 1}$ is the inverse function of the cumulative distribution of the random variable $\tilde{x}_{j}$.

38 $$ x_{ij} = F_{{x_{i} }}^{ - 1} \,(s_{ij} )\quad \forall i,j $$

After describing the robust optimization approach of the paper, first, the notation of the model is introduced as below.

## The main model

**Table:** 

Sets and Indices	Description
${\rm P}$	Set of eligible elective patients
$D$	Set of working days in the planning horizon
$O$	Set of operating rooms
*S*	Set of all available surgeons
*A*	Set of post-anesthesia resources
$\Psi$	Set of all scenarios
*H*	The set of working days in which, the surgery of elective patients is allowed
*p*	Index of elective patients ($p \in P$)
$t,\,\tau$	Index of working days ($t,\tau \in D$)
*s*	Index of surgeons ($s \in S$)
*o*	Index of operating rooms ($o \in O$)
$\Gamma_{s}$	Set of elective patients of surgeon *s*
$a,\,\alpha$	Indexes of post-anesthesia resources (*a* = 1 for ICU units and *a* = 2 for post-anesthesia beds)
$\Omega ,\,\Omega^{\prime }$	Index of scenarios

**Table:** 

Parameters	Description
$F_{t}^{c}$	The cost of activating each operating room in the ward of COVID-19 patients in day *t*
$F_{t}^{nc}$	The cost of activating each operating room in the ward of non-COVID-19 patients in day *t*
$B_{t}$	The regular operating hours of each operating room and surgeon in day *t*
$MO_{ot}$	The maximum overworking time of operating room *o* in day *t*
$d_{p}$	The duration time for surgery of elective patient *p* (sum of set up time, operating time and clean up time)
$dE_{t\Omega }^{nc}$	The duration time required for surgery of non-COVID-19 emergency patients under scenario $\Omega$
$dE_{t\Omega }^{c}$	The duration time required for surgery of COVID-19 emergency patients under scenario $\Omega$
$\rho_{p}$	Health status of patient *p*
$\alpha_{p}$	The preferred day for surgery of patient *p* set by the related surgeon
$\kappa_{1}$	The waiting cost per each day per each operated patient in the planning horizon
$\kappa_{2}$	The waiting cost per each day per each non-operated patient in the planning horizon
$W_{s}^{c}$	The wage cost of surgeon *s* per each day in the ward of COVID-19 patients
$W_{s}^{nc}$	The wage cost of surgeon *s* per each day in the ward of COVID-19 patients
$OW_{t}^{c}$	The overworking cost of each surgeon per hour in the ward of COVID-19 patients in day *t*
$OW_{t}^{nc}$	The overworking cost of each surgeon per hour in the ward of non-COVID-19 patients in day *t*
$UW_{t}^{c}$	The idle cost of each surgeon per hour in the ward of COVID-19 patients in day *t*
$UW_{t}^{nc}$	The idle cost of each surgeon per hour in the ward of non-COVID-19 patients in day *t*
$U_{s}$	The maximum days that surgeon *s* can attend in the hospital
$L_{s}$	The minimum days that surgeon s should attend in the hospital
$R_{a}$	The number of resource *a*
$RD_{pa}$	The number of days that resource *a* is required by patient *p*
$p_{\Omega }$	The probability of scenario $\Omega$
$OO_{ot}^{c}$	The over-utilization cost of operating room *o* per hour in the ward of COVID-19 patients in day *t*
$OO_{ot}^{nc}$	The over-utilization cost of operating room *o* per hour in the ward of non-COVID-19 patients in day *t*
$UO_{t}^{c}$	The under-utilization cost of each operating room per hour in the ward of COVID-19 patients
$UO_{t}^{nc}$	The under-utilization cost of each operating room per hour in the ward of non-COVID-19 patients
$OR_{at}^{c}$	The over-utilization cost of resource *a* per hour in the ward of COVID-19 patients in day *t* per hour
$OR_{at}^{nc}$	The over-utilization cost of resource *a* per hour in the ward of non-COVID-19 patients in day *t* per hour
$UR_{at}^{c}$	The under-utilization cost of resource *a* per hour in the ward of COVID-19 patients in day *t* per hour
$UR_{at}^{nc}$	The under-utilization cost of resource *a* per hour in the ward of non-COVID-19 patients in day *t* per hour
$RE_{at\Omega }^{c}$	The number of resource *a* requested by COVID-19 patients in day *t* under scenario $\Omega$
$RE_{at\Omega }^{nc}$	The number of resource *a* requested by non-COVID-19 patients in day *t* under scenario $\Omega$
$OUI_{o}^{nc}$	The over-utilization index of operating rooms in the ward of non-COVID-19 patients
$UUI_{o}^{nc}$	The under-utilization index of operating rooms in the ward of non-COVID-19 patients
$OUI_{o}^{c}$	The over-utilization index of operating rooms in the ward of COVID-19 patients
$UUI_{o}^{c}$	The under-utilization index of operating rooms in the ward of COVID-19 patients
$OUIPA_{a}^{nc}$	The over-utilization index of resource *a* in the ward of non-COVID-19 patients
$UUIPA_{a}^{nc}$	The under-utilization index of resource *a* in the ward of non-COVID-19 patients
$OUIPA_{a}^{c}$	The over-utilization index of resource *a* in the ward of COVID-19 patients
$UUIPA_{a}^{c}$	The under-utilization index of resource *a* in the ward of COVID-19 patients

**Table:** 

Variables	Description
$x_{pot\Omega }$	The binary variable with value 1 if elective patient *p* is assigned to the operating room *o* under scenario $\Omega$ and 0 otherwise
$v_{p\Omega }$	The binary variable with value 1 if elective patient p is not operated in scenario $\Omega$
$Q_{o}$	The binary variable with value 1 if operating room *o* is assigned to the ward of emergency COVID-19 patients and 0 otherwise
$Q_{ot\Omega }^{c}$	The binary variable with value 1 if operating room *o* is activated and assigned to the ward of emergency COVID-19 patients in day *t* under scenario $\Omega$.and 0 otherwise
$Q_{ot\Omega }^{nc}$	The binary variable with value 1 if operating room *o* is activated and assigned to the ward of emergency non-COVID-19 patients in day *t* under scenario $\Omega$.and 0 otherwise
$vdE_{ot\Omega }^{nc}$	The duration of surgery of non-COVID-19 emergency patients in operating room *o* under scenario $\Omega$
$vdE_{ot\Omega }^{c}$	The duration of surgery of COVID-19 emergency patients in operating room *o* under scenario $\Omega$
$Z_{st}^{c}$	The binary variable with value 1 if surgeon *s* attend in hospital and the ward of emergency COVID-19 patients in day *t* and 0 otherwise
$Z_{st}^{nc}$	The binary variable with value 1 if surgeon *s* attend in hospital and the ward of non-COVID-19 patients in day *t* and 0 otherwise
$RE_{a}$	The number of resources *a* allocated to the COVID-19 emergency patients

Based on the above notations and description, the scenario-based robust optimization model of the paper is as the following:

39 $$ \begin{aligned} Min\,z & = \sum\limits_{\Omega \in \Psi } {p_{\Omega } } .\,z_{\Omega } + \lambda .\,\sum\limits_{\Omega \in \Psi } {p_{\Omega } } \cdot \left( {\sum\limits_{o \in O} {\sum\limits_{t \in D} {\left( {OO_{ot}^{nc} .\,\varepsilon 1_{ot\Omega }^{ - } + UO_{ot}^{nc} .\,\varepsilon 1_{ot\Omega }^{ + } } \right)} } } \right. \\ & \quad + \sum\limits_{o \in O} {\sum\limits_{t \in D} {\left( {OO_{ot}^{c} \cdot \,\varepsilon 2_{ot\Omega }^{ - } + UO_{ot}^{c} \cdot \,\varepsilon 2_{ot\Omega }^{ + } } \right)} } + \sum\limits_{t \in D} {\left( {OW_{t}^{nc} \cdot \,\varepsilon 3_{t\Omega }^{ - } + UW_{t}^{nc} \cdot \,\varepsilon 3_{t\Omega }^{ + } } \right)} \\ & \quad + \sum\limits_{t \in D} {\left( {OW_{t}^{c} \cdot \,\varepsilon 4_{t\Omega }^{ - } + UW_{t}^{c} \cdot \,\varepsilon 4_{t\Omega }^{ + } } \right)} + \sum\limits_{a \in A} {\sum\limits_{t \in D} {\left( {OR_{at}^{nc} \cdot \,\varepsilon 5_{at\Omega }^{ - } + UR_{at}^{nc} \cdot \,\varepsilon 5_{at\Omega }^{ + } } \right)} } \\ & \quad + \left. {\sum\limits_{a \in A} {\sum\limits_{t \in D} {\left( {OR_{at}^{c} \cdot \,\varepsilon 6_{at\Omega }^{ - } + UR_{at}^{c} \cdot \,\varepsilon 6_{at\Omega }^{ + } } \right)} } } \right) \\ &\quad + \delta \cdot \,\sum\limits_{\Omega \in \Psi } {p_{\Omega } \cdot \,\left( {z_{\Omega } - \sum\limits_{{\Omega^{\prime } \in \Psi }} {p_{{\Omega^{\prime } }} \cdot \,z_{{\Omega^{\prime } }} } + 2\theta_{\Omega } } \right)} \\ \end{aligned} $$

40 $$ \begin{array}{*{20}l} {\text{s.t.}} \hfill \\ {z_{\Omega } = \sum\limits_{o \in O} {\sum\limits_{t \in D} {\left( {F_{t}^{c} \,.\,\,Q_{ot\Omega }^{c} + F_{t}^{nc} \,.\,\,Q_{ot\Omega }^{nc} } \right)} } + \sum\limits_{s \in S} {\sum\limits_{t \in D} {\left( {W_{s}^{c} .\,Z_{st}^{c} + W_{s}^{nc} \,.\,\,Z_{st}^{nc} } \right)} } } \hfill \\ { + \sum\limits_{o \in O} {\sum\limits_{p \in P} {\sum\limits_{t \in D} {\kappa_{1} \left[ {\rho_{p} (t - \alpha_{p} )\,\,.\,x_{pot\Omega } } \right]} } } + \sum\limits_{p \in P} {\kappa_{2} \left[ {\rho_{p} (|D| + 1 - \alpha_{p} )\,.\,v_{p\Omega } } \right]} } \hfill \\ \end{array} $$

41 $$ z_{\Omega } - \sum\limits_{\Omega ^{\prime} \in \Psi } {p_{\Omega ^{\prime}} z_{\Omega ^{\prime}} } + \theta_{\Omega } \ge 0\quad \forall \Omega \in \Psi $$

42 $$ \sum\limits_{o \in O} {\sum\limits_{t \in H} {x_{pot\Omega } } } + v_{p\Omega } = 1\quad \forall p \in P,\Omega $$

43 $$ \sum\limits_{o \in O} {\sum\limits_{{p\, \in \Gamma_{s} }} {d_{p} .x_{pot\Omega } } } \le B_{t} .\,Z_{st}^{nc} \quad \forall t \in D,\,s \in S,\,\Omega \in \Psi $$

44 $$ \sum\limits_{o \in O} {vdE_{ot\Omega }^{nc} } = dE_{t\Omega }^{nc} \quad \forall t \in D,\,\Omega \in \Psi $$

45 $$ \sum\limits_{o \in O} {vdE_{ot\Omega }^{c} } = dE_{t\Omega }^{c} \quad \forall t \in D,\,\Omega \in \Psi $$

46 $$ \sum\limits_{p\, \in P} {d_{p} .x_{pot\Omega } } + vdE_{ot\Omega }^{nc} + \varepsilon 1_{ot\Omega }^{ + } - \varepsilon 1_{ot\Omega }^{ - } = B_{t} .\,Q_{ot\Omega }^{nc} \quad \forall o \in O,\,t \in D,\,\Omega \in \Psi $$

47 $$ \varepsilon 1_{ot\Omega }^{ - } \le MO_{ot} \quad \forall o \in O,\,t \in D,\,\Omega \in \Psi $$

48 $$ vdE_{ot\Omega }^{c} + \varepsilon 2_{ot\Omega }^{ + } - \varepsilon 2_{ot\Omega }^{ - } = B_{t} .\,Q_{ot\Omega }^{c} \quad \forall o \in O,\,t \in D,\,\Omega \in \Psi $$

49 $$ \varepsilon 2_{ot\Omega }^{ - } \le MO_{ot} \quad \forall o \in O,\,t \in D,\,\Omega \in \Psi $$

50 $$ \sum\limits_{o \in O} {\sum\limits_{p\, \in P} {d_{p} .\,x_{pot\Omega } } } + \sum\limits_{o} {vdE_{ot\Omega }^{nc} } + \varepsilon 3_{t\Omega }^{ + } - \varepsilon 3_{t\Omega }^{ - } = B_{t} .\,\sum\limits_{s \in S} {Z_{st}^{nc} } \,\,\,\,\,\forall t \in D,\,\Omega \in \Psi $$

51 $$ \sum\limits_{o \in O} {vdE_{ot\Omega }^{c} } + \varepsilon 4_{t\Omega }^{ + } - \varepsilon 4_{t\Omega }^{ - } = \sum\limits_{s \in S} {B_{t} .\,Z_{st}^{c} } \,\,\,\,\,\,\,\,\,\,\,\,\,\,\,\,\,\,\,\forall t \in D,\,\Omega \in \Psi $$

52 $$ Z_{st}^{c} + Z_{st}^{nc} \le 1\quad \forall s \in S,\,t \in D $$

53 $$ L_{s} \le \sum\limits_{s \in S} {(Z_{st}^{c} + Z_{st}^{nc} )} \le U_{s} \quad \forall s \in S $$

54 $$ Q_{ot\Omega }^{c} \le Q_{o} \quad \forall o,t,\Omega $$

55 $$ Q_{ot\Omega }^{nc} \le 1 - Q_{o} \quad \forall o,t,\Omega $$

56 $$ \varepsilon 1_{ot\Omega }^{ + } \le M.\,Q_{ot\Omega }^{nc} \quad \forall o \in O,\,t \in D,\,\Omega \in \Psi $$

57 $$ \varepsilon 1_{ot\Omega }^{ - } \le M.\,Q_{ot\Omega }^{nc} \quad \forall o \in O,\,t \in D,\,\Omega \in \Psi $$

58 $$ \varepsilon 2_{ot\Omega }^{ + } \le M.\,Q_{ot\Omega }^{c} \quad \forall o \in O,\,t \in D,\,\Omega \in \Psi $$

59 $$ \varepsilon 2_{ot\Omega }^{ - } \le M.\,Q_{ot\Omega }^{c} \quad \forall o \in O,\,t \in D,\,\Omega \in \Psi $$

60 $$ \sum\limits_{p \in P} {\sum\limits_{o \in O} {\sum\limits_{{\tau = t + \sum\limits_{\alpha = 1}^{a - 1} {RD_{p\alpha } } }}^{{t + \sum\limits_{\alpha = 1}^{a} {RD_{p\alpha } } - 1}} {x_{pot\Omega } } } } + RE_{at\Omega }^{nc} + \varepsilon 5_{at\Omega }^{ + } - \varepsilon 5_{at\Omega }^{ - } = R_{a} - RE_{a} \quad \forall a \in A,\,t \in D,\,\Omega \in \Psi $$

61 $$ RE_{at\Omega }^{c} + \varepsilon 6_{at\Omega }^{ + } - \varepsilon 6_{at\Omega }^{ - } = RE_{a} \quad \forall \,a \in A,\,\,t \in D,\Omega \in \Psi $$

62 $$ \varepsilon 1_{ot\Omega }^{ + } ,\,\varepsilon 1_{ot\Omega }^{ - } ,\,\varepsilon 2_{ot\Omega }^{ + } ,\,\varepsilon 2_{ot\Omega }^{ - } \in \{ 0,\,1\} \quad \forall o \in O,t \in D,\Omega \in \Psi $$

63 $$ \varepsilon 3_{t\Omega }^{ + } ,\,\,\varepsilon 3_{t\Omega }^{ - } ,\,\,\varepsilon 4_{t,\Omega }^{ + } ,\,\,\varepsilon 4_{t,\Omega }^{ - } ,\,\varepsilon 5_{t\Omega }^{ + } ,\,\,\varepsilon 5_{t\Omega }^{ - } ,\,\varepsilon 6_{t\Omega }^{ + } ,\,\varepsilon 6_{t\Omega }^{ - } \ge 0\quad \forall t \in D,\,\Omega \in \Psi $$

64 $$ Q_{ot\Omega }^{c} ,\,\,Q_{ot\Omega }^{nc} ,\,Q_{o} \in \{ 0,1\} \quad \forall o \in O,\,t \in D,\,\Omega \in \Psi $$

65 $$ Z_{st}^{c} ,\,Z_{st}^{nc} \in \{ 0,1\} \quad \forall s \in S,\,t \in D $$

66 $$ x_{pot\Omega } ,\,v_{p\Omega } \in \{ 0,\,1\} \quad \forall t \in D,\,\Omega \in \Psi $$

67 $$ vdE_{ot\Omega }^{nc} ,\,vdE_{ot\Omega }^{c} \ge 0\quad \forall o \in O,\,t \in D,\,\Omega \in \Psi $$

68 $$ RE_{a} \ge 0\quad \forall a \in A $$

Equation 39 shows the objective function of the model comprised of the expected value of costs, the penalties regarded for overworking and idle time of surgeons, the over-utilization and under-utilization costs of operating rooms in both the COVID-19 and non-COVID-19 wards, the over-utilization and under-utilization costs of post-anesthesia resources in both the COVID-19 and non-COVID-19 wards as well as the cost considered for the deviation in the solution for different scenarios. Equation 40 stipulates the cost of the model for each scenario, including the activation costs of operating rooms for non-COVID-19 and COVID-19 patients, the wage costs of surgeons, and the waiting costs of elective patients. Equation 41 is used for the linearization of the model as expected earlier. Equation 42 specifies whether an elective patient is operated on the planning horizon or not. Also, regarding this equation, the surgery of elective patients is only restricted to the days of set *H*. Based on Eq. 43, the surgery duration of elective patients related to each surgeon should not exceed the regular time that surgeon is in the hospital. Equations 44 and 45 respectively indicate that the sum of the surgery duration of the non-COVID-19 and COVID-19 emergency patients in the associated operation rooms should be equal to the required duration of them as realized in each scenario. Equation 46 shows that the duration of surgery for non-COVID-19 elective and emergency patients in an operating room should not exceed the available regular time in that operating room unless some surgeries are performed during overtime. However, the overtime duration in each day could not be more than the maximum allowable duration, as illustrated in Eq. 47. The same argument is true for the operation rooms assigned to COVID-19 patients (Eqs. 48, 49). Equations 50 and 51 are used to set the overworking time of surgeons attended in the operating rooms of non-COVID-19 and COVID-19 patients, respectively. Equation 52 denotes that a surgeon in a day is only allowed to be employed in the COVID-19 operating rooms or the non-COVID-19 ones. Equation 53 indicates that the number of days a surgeon must be in the hospital cannot be less than the minimum number and more than the maximum number specified in its contract. According to Eq. 54, an operating room could be activated in a day for the COVID-19 patients if it is assigned to the COVID-19 patients in the first stage, i.e., the beginning of the planning horizon. The same holds for non-COVID-19 patients as shown in Eq. 55. Equations 56–59 impose that the slack and surplus variables of the operating rooms in each day and each scenario will be equal to zero if the related operating room is inactive in that day and that scenario. Equations 60 and 61 limit the usage of post-anesthesia resources to their standard capacity unless they are used over-utilization. Finally, Eqs. 62–68 describe the decision variable of the model.

## Case study

The case study of the paper is about the Orthopedic Surgery department in one of the public hospitals in Tehran, the capital of Iran. This department carries out the surgery of knee arthroscopy and meniscectomy, scapular arthroscopy and decompression, carpal tunnel treatment, knee arthroscopy, etc. Also, emergency patients in this ward are usually patients who suffer from fractures in the limbs, chest, etc., resulting from a car accident or workplace injuries. This department has six operating rooms, 12 ICU beds, and 30 post-anesthesia beds. For this case, the planning horizon is two weeks with 14 days, and the elective surgeries only are performed on days 1–5 and 8–13, which means the first five days of each week. It is notable that in reality, if some scenarios entail the necessity of elective surgery on the weekends (the last two days of a week), the duration of that surgery is considered as overtime. The department is in contract with six surgeons whose minimum and maximum days of their attendance in the hospital are as Table 2.

**Table 2:** The minimum and maximum days of surgeons in the hospital in the planning horizon

Surgeons	The minimum number of days	The maximum number of days
1	3	6
2	3	5
3	3	6
4	4	6
5	3	4
6	2	3

The information about the elective patients of surgeons is according to Table 3. This information contains the health status of patients, the appropriate time for the surgery of each patient, and the number of hospital resources consumed by each patient. The information also reflects the desired time block of surgeons for attending hospital and performing the surgery of patients. Due to considering penalties for the deviation from the suggested time of surgeons, the proposed model aims to propose a timetable that satisfies the surgical teams as much as possible. From Table 3, it is also evidence that each patient will stay in the hospital for a maximum of 5 days from its arrival to its departure. Therefore, the periods of the model are extended from *T* to *T* + 4, i.e., *t* = 1, 2…, *T* + 4.

**Table 3:** The information related to the surgery of elective patients

Patient (*p*)	Surgeon (*s*)	$\alpha_{p}$	$\rho_{p}$	$RD_{p1}$	$RD_{p2}$	$d_{p}$ (h)
1	1	3	3	1	3	3
2	1	3	4	2	3	3.5
3	1	5	3	0	3	2.5
4	1	5	3	0	3	2.5
5	1	5	3	0	3	3
6	2	4	3	1	4	3
7	2	4	3	0	4	2.5
8	2	4	3	0	4	3
9	2	8	3	1	3	3
10	2	8	3	0	4	3
11	3	2	3	0	4	3
12	3	2	4	2	3	3.5
13	3	5	3	1	3	3
14	3	5	3	1	3	2.5
15	3	5	3	0	3	3
16	4	1	5	2	3	4
17	4	1	5	2	3	4
18	4	2	5	2	3	4
19	4	2	3	0	4	3
20	4	10	5	2	3	4
21	5	1	4	2	3	3
22	5	4	3	1	3	3
23	5	9	3	1	3	2.5
24	5	9	3	0	4	2.5
25	5	9	3	1	3	2.5
26	6	10	5	2	3	4
27	6	10	4	2	3	3
28	6s	12	5	2	3	4

The regular hours of operating rooms are 8 h/day. The opening cost of operating rooms in the non-COVID-19 ward is about 1000 $/day, while that cost in the COVID-19 ward will be 1500 $/day. The overworking costs of each operating room per hour in the non-COVID-19 ward are 150 $/h, while that cost in the COVID-19 ward is 250 $/h. For the emergency patient, the Poisson distribution with arrival rate 8 patients per day ($$\lambda = 6$$, where $$\lambda$$ is the parameter of Poisson distribution in the form of $$f(k;\lambda ) = \lambda^{k} e^{ - \lambda } /k!$$). Based on historical data, it is assumed that the probability that an emergency patient has COVID-19 disease is 10%. After simulating the number of emergency patients each day, the log-normal distribution with a Mean of 3 h and standard deviation of 0.5 is used to generate the surgery duration of each emergency patient. Summing up the durations of all emergency patients, the total duration required for surgery in the operating rooms is determined. Moreover, the length of stay in days that each emergency patient occupies an ICU bed follows a discrete probability distribution with the probabilities shown in Table 4.

**Table 4:** The probability of length of stay in days for each emergency patient

Number of days	0	1	2	3	4
Probability	0.1	0.15	0.65	0.05	0.05

The overtime cost of surgeons per hour is 50 $, while the wage of each surgeon team per day is 530 $/day. The overworking and wage of surgeons assigned to the COVID-19 operating rooms is considered 1.5 times more than surgeons in the ward of non-COVID-19 patients. The maximum overworking time of each operating room is set to 4 h, i.e., the maximum duration is required for operating a patient with bad health status. The over-utilization cost of each ICU bed is regarded as equal to 500 $/day (Jebali and Diabat 2017). This cost for each post-anesthesia bed is 50 $/day whilst some studies neglect it (Jebali and Diabat 2017). Again, the cost of resources in the COVID-19 ward is 1.5 times more than that of the non-COVID-19 ward. Finally, $$\kappa_{1}$$ = 50 $ and $$\kappa_{2}$$ = 50 $ are considered for the waiting costs of related patients. Furthermore, due to the need for a high level of health services, the idle time cost of surgeons, as well as the under-utilization costs of operating rooms and post-anesthesia beds, is set equal to zero intentionally. However, in non-COVID-19 situations where the use of significant resources is not necessary, considering under-utilization costs might yield a more efficient solution in terms of costs, although this is not the case in this paper.

To implement the SAA method, the parameters of the algorithm are considered as *L* = 5, 10, 20, and 50 and 100; *M* = 10 and *L′* = 200. Regarding the SAA parameters and the value of the parameters in the considered problem, the results of the model implementation for different values of *L* have been listed in Table 5. In this table, *LB* and *UB* denote respectively the lower bound and upper bound of the model solution, *GAP* indicates the difference between the lower bound and upper bound in percent, and CPU (s) shows the time to solve the model in second. Furthermore, it should be pointed out that all numerical experiments in this section are conducted using GAMS 23.0 software using CPLEX solver in a PC with AMD Ryzen 7 3700U @4 GHz with a memory of 16.0 GB.

**Table 5:** The results of the SAA algorithm for different values of L

Sample size L	Number of Binary variables		Number of all variables		Number of constraints		LB	UB	GAP (%)	CPU (s)	
	Min	Max	Min	Max	Min	Max				Min	Max
5	9842	384,800	20,895	827,025	5851	229,331	101,227	106,732	5.44	109	15,275
10	19,462	384,800	41,565	827,025	11,581	229,331	97,156	99,333	2.24	263	15,339
20	38,702	384,800	82,905	827,025	23,041	229,331	95,478	97,080	1.67	638	15,342
50	96,422	384,800	206,925	827,025	57,421	229,331	95,644	96,563	0.96	1962	15,532
100	192,622	384,800	413,625	827,025	114,721	229,331	95,986	96,497	0.53	6308	15,632

The results in Table 5 are evident that by increasing the sample size (*L*), the optimality gap is decreased, the numbers of variables and constraints are increased, and the CPU time is increased, as expected. For further analysis, *L* = 50 is considered due to its relatively short CPU time and low gap. For this sample size, although the lower bound as the final solution has the objective value equal to 95,644, however, the average cost without constraints violation penalties is 72,375. This value will be used for the analysis of robust solutions later.

The timetabling program of the surgical team in the planning horizon also is determined according to Table 6. Moreover, based on the model results and considering the probability of different scenarios, the average number of elective patients who are operated on in the planning horizon is 23.7 of 28 available patients, and therefore the rejection rate is relatively low. Also, in the solution, one operating room, three anesthesia beds, and two ICU units should be assigned to COVID-19 patients.

**Table 6:** The timetable of surgeons in the planning horizon

Surgeon	Ward	1	2	3	4	5	6	7	8	9	10	11	12	13	14	sum
1	Non-COVID-19	*					*						*			6
	COVID-19		*									*		*		
2	Non-COVID-19	*							*						*	5
	COVID-19			*									*			
3	Non-COVID-19					*				*						6
	COVID-19	*					*	*	*				*			
4	Non-COVID-19	*	*					*				*				6
	COVID-19									*	*					
5	Non-COVID-19			*							*					4
	COVID-19				*	*										
6	Non-COVID-19				*									*		3
	COVID-19														*	

A remarkable note is about the utilization rate of the hospital facilities such as operating rooms and post-anesthesia beds. The utilization rate could be defined as the duration a facility is used over its total available duration. However, according to surgical standards, operating rooms and their equipment should be used in one shift per day, as far as possible. Therefore, by this standard, we consider the effective utilization rate of hospital equipment as the sum of the duration of all active facilities in the planning horizon is used per day over the total duration of one shift. In this paper, the sum of the idle time of facilities regarding the probability of the scenarios is considered as the index for the under-utilization rate of facilities. On the other hand, some of the overworking times of facilities will denote the over-utilization index of facilities. Hereafter, Eqs. 69 and 70 are used for indicating the over-utilization and under-utilization indexes of operating rooms in the ward of non-COVID-19 patients, while Eqs. 71 and 72 are used for representing those indexes for the operating rooms in the ward of COVID-19 patients, respectively. Based on the optimal solution and Eqs. 69–72, the over-utilization and under-utilization indexed of operating rooms in the COVID-19 and non-COVID-19 ward are obtained as $OUI_{o}^{nc} = 38.83$, $UUI_{o}^{nc} = 3.56$, $OUI_{o}^{c} = 0$ and $UUI_{o}^{c} = 42$.

69 $$ OUI_{o}^{nc} = \sum\limits_{\Omega \in \Psi } {\sum\limits_{o \in O} {\sum\limits_{t \in D} {p_{\Omega } .\,\varepsilon 1_{ot\Omega }^{ - } } } } $$

70 $$ UUI_{o}^{nc} = \sum\limits_{\Omega \in \Psi } {\sum\limits_{o \in O} {\sum\limits_{t \in D} {p_{\Omega } .\,\varepsilon 1_{ot\Omega }^{ + } } } } $$

71 $$ OUI_{o}^{c} = \sum\limits_{\Omega \in \Psi } {\sum\limits_{o \in O} {\sum\limits_{t \in D} {p_{\Omega } .\,\varepsilon 2_{ot\Omega }^{ - } } } } $$

72 $$ UUI_{o}^{c} = \sum\limits_{\Omega \in \Psi } {\sum\limits_{o \in O} {\sum\limits_{t \in D} {p_{\Omega } .\,\varepsilon 2_{ot\Omega }^{ + } } } } $$

The same argument could be applied for utilization rates of post-anesthesia resources, i.e., post-anesthesia beds and ICU units. For the over-utilization and under-utilization indexes of these resources, Eqs. 73–76 are proposed. It should be pointed out that in the cases of over-utilization of the operating rooms, the use of operating rooms in the overworking hours could be applied though this is not preferable. However, the shortage of post-anesthesia beds and ICUs are not easily remedied, and in scenarios where there is a shortage of these units, the proposed schedule may run into serious problems. One could set large values for the penalties of the shortage in such resources to avoid resource violation in the solution. However, this viewpoint is a pessimistic one, and when the probability for the occurrence of acute scenarios is low, the pessimistic solution imposes high costs to hospitals and might be undesirable by hospital managers. Indeed, a robust solution could provide a trade-off between solution costs and the level of constraints violations. In the proposed model, using the sensitivity analysis on the penalty cost of post-anesthesia resources, the effects of robustness in the solutions are discussed in the next section.

73 $$ OUIPA_{a}^{nc} = \sum\limits_{\Omega \in \Psi } {\sum\limits_{t \in D} {p_{\Omega } .\,\varepsilon 5_{at\Omega }^{ - } } } $$

74 $$ UUIPA_{a}^{nc} = \sum\limits_{\Omega \in \Psi } {\sum\limits_{t \in D} {p_{\Omega } .\,\varepsilon 5_{at\Omega }^{ + } } } $$

75 $$ OUIPA_{a}^{c} = \sum\limits_{\Omega \in \Psi } {\sum\limits_{t \in D} {p_{\Omega } .\,\varepsilon 6_{at\Omega }^{ - } } } $$

76 $$ UUIPA_{a}^{c} = \sum\limits_{\Omega \in \Psi } {\sum\limits_{t \in D} {p_{\Omega } .\,\varepsilon 6_{at\Omega }^{ + } } } $$

Taking into account Eqs. 73–76, the utilization indexes of post-anesthesia resources in the robust solution are obtained as Table 7. These indexes do not indicate the acute status of the shortage of hospital resources on average. However, the change in each of the patient entry rates in pandemic conditions; For example, increasing the percentage of COVID-19 patients; can aggravate the situation so that a severe shortage of hospital resources leads to a high rate of patient rejection in reality and even the hospital will not be able to accept emergency patients.

**Table 7:** The utilization indexes of hospital resource

Index	Resource	
	Post-anesthesia beds	ICU units
$OUIPA_{a}^{nc}$	7.1	8.4
$UUIPA_{a}^{nc}$	67.4	99.6
$OUIPA_{a}^{c}$	0.2	1.9
$UUIPA_{a}^{c}$	21.1	21.3

For validation purposes, the results of the model could be compared with that of alternative ones. One of the common models available in the literature is the model in which the average value of uncertain parameters is applied for the value of these parameters. This approach is similar to the case where there is only one scenario with the mentioned values for uncertain parameters in the problem. Although by solving such a model, it is possible to determine both the value of the first stage variables and the second stage variables for the mentioned scenario, but only the results of the first variables can be implemented in practice. In other words, this model is used to determine the value of the first-stage variables that must be assigned before uncertain events occur. Then, the first stage variables are considered constant and the second stage ones are determined according to the scenario that occurs in reality and the solution of the related model for that scenario. In the literature of stochastic programming models, the mentioned alternative solution is called “the expected value solution” or briefly, EV (Kail & Mayer, 2005). Moreover, the resulting solution of the proposed model is referred to as the “Here-And-Now solution” which instead of simply determining the value of the first stage variables for the average value of uncertain parameters, attempts to determine them such that the expected value of the objective function for the uncertain scenarios over uncertain parameters that might be occurred in reality is optimized (Kail & Mayer, 2005).

It was shown that the “Here-And-Now solution” outperforms the “EV” solution; as an alternative model; and this indicates by self the validity of the scenario-based stochastic programming models (Kail & Mayer, 2005). In this regard and by referring to Table 5, the proposed model has the objective function value equal to 96,497 for a large sample of 100 scenarios. However, if the EV solution is used for determining the first-stage variables of the problem and then the optimal value of the second-stage variable, as well as the objective function, is identified for each scenario, the average value of the objective function over all scenarios will be 109,837. This shows that the proposed model improves the objective function by 109,837–96,497 = 13,340. So, the validity of the proposed model in comparison with alternative approaches can be acknowledged from this perspective.

After describing the original results of the model, in the next sub-sections, first, the changes in the robust parameters are evaluated, and second, it is shown how changes in the model parameters could affect the proposed solutions.

### Changes in the robust parameters

**Fig. 2:**
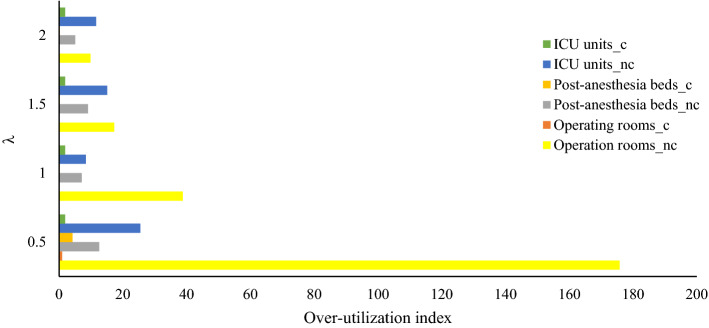
Over-utilization indexes of hospital facilities versus penalty cost of constraints

**Fig. 3:**
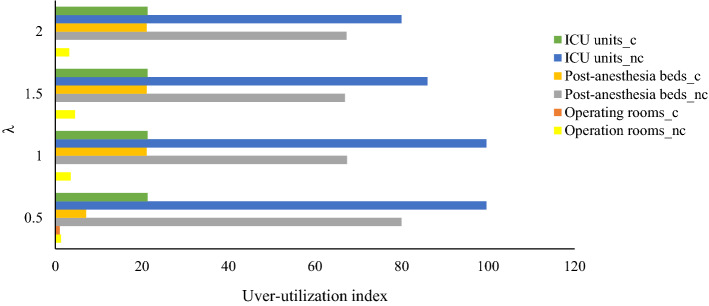
Under-utilization indexes of hospital facilities versus penalty cost of constraints

As one of the main aims of the robust optimization approaches, making a trade-off between model feasibility and model optimality is investigated in this section. To do so, the penalties considered for the violation of constraints are increased, and their effects on the average costs without and with penalties in the objective function as well as the utilization indexes of facilities are discussed. Four levels are considered for the penalty, including $\lambda$ = 0.5, 1, 1.5, and 2. Table 8, represents the average costs of the robust solution for different values of $\lambda$. Figures 2 and 3 show the over-utilization and underutilization indexes of hospital facilities for different values of $\lambda$ respectively. In these figures, the subscripts "c" and "nc" have been used to denote the facilities in the COVID-19 and non-COVID-19 wards, respectively.

**Table 8:** The results of the robust optimization model versus penalty cost of constraints

Penalty ($\lambda$)	Average cost (C2) (without penalty of constraints violation)	Robust objective function (C3)	Penalty cost (C3-C2)	Average number of elective patients operated in the planning horizon
0.5	53,500	78,098	24,598	25
1	72,375	95,644	23,269	23.5
1.5	80,070	102,435	22,365	21.1
2	85,650	107,320	21,670	20.1

As Table 8 illustrates, increasing the penalty cost of the constraints results in more conservative solutions with more average costs while decreasing the constraints violation and consequently decreasing the penalty cost. Moreover, based on the results in Figs. 2, 3 and Table 8, more conservative solutions lead to better utilization of resources by decreasing the over-utilization and under-utilization rate of resources. However, the optimistic solutions, i.e., solutions by applying less value for $\lambda$, the rejection rate of elective patients as well as the average costs, are less, so these solutions might be more desirable by risk-averse managers. Therefore, the hospital managers could balance the mentioned consideration and set the penalty coefficient $\lambda$ at a level that balances between the optimality and desirability of solutions in terms of feasibility as well as resource utilization rate.

### Changes in the average of COVID-19 patients

One of the concerns in pandemics, such as COVID-19 disease, is the increasing trend in the number of patients, which puts a lot of pressure on medical staff and hospitals. Also, in the case of excessive exacerbation of the disease, it has been observed that many hospitals do not have the necessary capacity to handle new and emergency patients. This, in turn, causes further harm to patients, and their disease becomes more acute due to non-admission in hospitals. The model of the present paper does not specifically study the treatment of COVID-19 disease patients, but the broader prevalence of the disease can lead to a similar trend in the arrival rate of emergency patients who suffered COVID-19 and were referred to the hospital to be treated. To evaluate the effect of such a trend on the model results, the probability that an emergency patient has COVID-19 is increased in the generated data of sample case and its effects on the allocation of capacity as well as the overuse of hospital resources in this section. Table (9) reports the average cost, the robust objective function, the utilization indices, the number of elective patients operated in the planning horizon, and the average over-working duration of surgeons for various probabilities that an emergency patient has COVID-19. It should be noted that the overworking duration of surgeons in the ward of non-COVID19 and COVID-19 patients are determined based on Eqs. 77 and 78, respectively.

77 $$ OWS^{nc} = \sum\limits_{\Omega \in \Psi } {\sum\limits_{t \in D} {p_{\Omega } .\,\varepsilon 3_{t\Omega }^{ - } } } $$

78 $$ OWS^{c} = \sum\limits_{\Omega \in \Psi } {\sum\limits_{t \in D} {p_{\Omega } .\,\varepsilon 4_{t\Omega }^{ - } } } $$

The results of Table 9 clearly confirm that by increasing the prevalence of pandemic, the shortage of hospital resources, excessive workload of medical staffs, overuse of hospital resources, and medical costs, in general, are increased. In response to these negative consequences, hospital resources must be increased in the shortest possible time so that hospitals can achieve the same level of performance as before and provide appropriate services to patients. However, the economic pressures on governments and health systems as a result of the pandemic are preventing hospitals from being equipped more adequately and quickly. In such a situation, along with the redoubled efforts of the medical staff, logistics, and operations research techniques, such as the model proposed in this paper, can help to properly allocate hospital resources and provide fair services to patients and victims. Nevertheless, along with all efforts, adherence to health protocols and prevention of disease transmission can prevent, more than anything, excessive erosion of hospital resources, the fatigue of medical staff, and better recovery of patients.

**Table 9:** The results of the robust optimization model versus the probability of COVID-19 disease in emergency patients

The probability of COVID-19 disease in emergency patients	0.1	0.3	0.6	0.9
Average cost	72,375	73,990	75,980	78,624
Robust objective function	95,644	94,455	102,689	125,041
Average number of elective patients operated in the planning horizon	23.7	21.8	20.6	18.4
$OUI_{o}^{nc}$	38.83	42.373	45.574	64.13
$OUI_{o}^{c}$	42	45.798	58.994	66.21
$OUIPA_{1}^{nc}$	7.1	9.4	12	15
$OUIPA_{2}^{nc}$	8.4	11.5	31.133	40.41
$OUIPA_{1}^{c}$	0.2	6.27	10.31	26.72
$OUIPA_{2}^{c}$	1.9	7	47.14	90.65
$OWS^{nc}$	25.34	54.49	125.24	166.23
$OWS^{c}$	1.26	2.08	52.74	86.92
Number of operating rooms assigned to COVID-19 ward	1	1	2	3
Number of post-anesthesia beds assigned to COVID-19 ward	3	4	16	22
Number of ICU units assigned to COVID-19 ward	2	2	7	11

## Conclusion

In this paper, the problems of scheduling operating room and patients' surgery and allocation of surgical teams to working days in pandemic conditions are studied by considering the resource constraints of hospitals. The considered resources included operating rooms, post-anesthesia beds, ICU units, and surgical teams. The proposed model, unlike many operating room scheduling models, attempts to determine the time blocks of surgical teams based on the hospital requirement instead of assuming the time blocks are predetermined. Also, as explained, the pandemic treatment protocols in pandemic conditions impose new restrictions on hospital programs and the ways patients are admitted to the hospital. Figure 1 showed the protocol used in the condition of COVID-19, which has been applied in the studied hospitals and many other hospitals around the world. According to this protocol, the COVID-19 patient ward must be separated from other hospital wards, and only emergency patients with COVID-19 can be admitted to the hospital. Other elective patients, with the necessity of surgery and no COVID-19 infection, should be appropriately scheduled for the surgery in the planning horizon, of course, if the limited resources of the hospital and surgical teams do not prevent it. The proposed model also assigned the resources of the hospital to the COVID-19 and non-COVID-19 wards as well as separated the time-block of surgeons to days that they should attend in the COVID-19 wards and the days they assigned to the non-COVID-19 ward. The results showed that this provision, which is by the treatment protocols, made the model more complex and imposed more restrictions on the optimal allocation of the hospital. Due to the uncertainty in the time required for surgery of emergency patients as well as their use of post-anesthesia beds and ICU units, scenario-based robust optimization was used to consider the uncertainty in the model. The robust optimization method, unlike the original stochastic planning model, made it possible to achieve more efficient and less conservative solutions by accepting a certain level of constraints violation, especially in critical scenarios with low probability. Also, the Sample Average Approximation (SAA) approach was used to achieve the optimal solution of the robust model due to the efficiency of this method in scenario-based models where computational complexity increases.

The proposed model was implemented in a hospital in Iran dedicated to surgeries related to bone and joint fractures. For this case, the scheduling planning of the hospital for a two-week planning horizon was extracted. To do so, proper sample size in the SAA method is stipulated, which resulted in a low gap between the upper and lower bound solution and reached the solution in proper time duration. Moreover, regarding the solution, over-utilization and under-utilization indices were defined for operating rooms as well as post-anesthesia beds and ICU units. Moreover, it was shown how the penalty cost of the robust optimization approach could affect the solutions in terms of the average cost and utilization indices. Representing the model results for different values of the robust parameter was stated that hospital managers, based on their risk-averse level, could select a solution that better balances the cost, constraints violation levels as well as utilization rates of hospital facilities. Furthermore, the effects of increasing the prevalence of pandemics on hospital planning were examined by changing the probability that an emergency patient suffers from COVID-19 disease. The results of the model clearly indicated that by growing the pandemic, the pressure on the hospital resources and the medical staff is increased, and due to insufficient hospital resources, in this case, some patients cannot receive adequate and efficient medical services. Based on this evidence, it was stated that better prevention against the disease could reduce the pressure on the health system and medical staff, especially in situations where it is not possible to increase hospital resources in the shortest possible time.

The results of the paper clearly exhibited the essential role of hospital resources in pandemic conditions, the lack of these resources with the growth of pandemics, and the need for their proper management to better respond to the patients and schedule surgical teams. However, the present study did not examine the relationship between hospitals and their coordination for capacity sharing. Undoubtedly, better information and resource sharing and disease control are essential in situations such as the COVID-19 epidemic, which could be a suggestion for future hospital planning and scheduling studies. Also, considering new assumptions and specific conditions of surgical teams in identifying their work schedule, such as limiting the number of days a surgical team can attend in the CODID-19 ward, which can affect hospital schedule in pandemic conditions, is also a suggestion for future studies. It should also be pointed out that although the COVID-19 protocol of the paper that was shown in Fig. 1 is a standard one and is adopted in many countries, the COVID-19 protocol and aspects of disease control in other hospitals and countries might be more complicated than it. For such protocols, more advanced models covering more aspects of planning; such as resource sharing; are necessary and proposed for future studies.
